# Identifying suspicious naevi with dermoscopy via variational autoencoder auxiliary generative classifiers

**DOI:** 10.1007/s13246-025-01636-9

**Published:** 2025-09-17

**Authors:** Fatima Al Zegair, Brigid Betz-Stablein, Monika Janda, H. Peter Soyer, Shekhar S. Chandra

**Affiliations:** 1 School of Electrical Engineering and Computer Science, The University of Queensland, Brisbane, QLD Australia; 2 Dermatology Research Centre, Frazer Institute, The University of Queensland, Brisbane, QLD Australia; 3 Centre for Health Services Research, Faculty of Health, Medicine and Behavioural Sciences, The University of Queensland, Brisbane, QLD Australia

**Keywords:** Suspicious naevi, Non-suspicious naevi, Deep convolutional generative adversarial network (DCGAN), Auxiliary classifier generative adversarial network (ACGAN), Variational autoencoder auxiliary classifier generative adversarial network (VAE-ACGAN), Manifold

## Abstract

A naevus is a benign melanocytic skin tumour made up of naevus cells, characterised by variations in size, shape, and colour. Understanding naevi is essential due to their significant role as markers for the risk of developing melanoma. This study focused on creating a visual representation called a manifold that illustrates the distribution of two types of naevi: suspicious and non-suspicious. The research aimed to classify real naevi using generative adversarial networks (GANs), while also generating realistic synthetic samples and interpreting their distribution through a variational manifold. This inquiry holds promise for applying data-driven methods for early melanoma detection by identifying distinct features linked with suspicious naevi. Our variational autoencoder auxiliary classifier generative adversarial network (VAE-ACGAN) for suspicious naevi revealed a manifold with outstanding performance, including specificity, sensitivity, and area under the curve (AUC) scores, particularly representing suspicious naevi. These models surpassed various deep learning frameworks in key performance metrics while producing a manifold that indicated a significant distinction between the two categories in the resultant image, yielding high-quality and life-like representations of naevi. The results highlight the potential application of GANs in expanding data sets and enhancing the effectiveness of deep learning algorithms in dermatology. Accurate identification and categorisation of naevi could facilitate early melanoma detection and deepen our understanding of these skin lesions through an interpretable clustering method based on visual similarities.

## Introduction

Skin cancer is the most common type of cancer in humans, which is typically detected through a visual inspection. The diagnostic process usually starts with an initial clinical screening, which is further complemented by additional techniques like dermoscopic analysis. If further confirmation is required, a biopsy is conducted, followed by histopathological examination to determine a definitive diagnosis [1]. Skin cancer can be categorised into three main types: basal cell carcinoma, squamous cell carcinoma, and melanoma, with melanoma being the most lethal form of skin cancer. Melanoma is exceptionally hazardous as it exhibits a swift and aggressive spread to lymph nodes, often before the cancer itself is even detected [2].

Timely and accurate detection of melanoma can significantly improve patients' survival rates. However, the manual identification of melanoma necessitates a considerable number of extensively trained specialists and is susceptible to variations in interpretation among different observers [3]. Consequently, the presence of automated computerised diagnostic systems for skin lesions becomes essential in order to provide valuable assistance and support to dermatologists in their decision-making process [4].

Computer-aided diagnosis (CAD) has gained significant attention as a prominent research area in medical imaging and diagnostic radiology [5]. CAD has found extensive application in the analysis of images across various medical fields, including the detection and diagnosis of skin cancer [6]. Nonetheless, accurately identifying melanoma remains a complex undertaking due to several factors. These factors include the limited contrast between lesions and the surrounding skin, the visual resemblance between melanomas and non-melanoma lesions, and the variations in skin characteristics such as colour, hair, and veins [3, 7].

Naevi are characterised as harmless groups of cells that undergo clonal proliferation and exhibit the melanocytic phenotype [8]. Extensive evidence has established a strong connection between benign naevi and melanomas. Furthermore, benign naevi are recognised as frequent mimics of melanoma, as the number of naevi represents the most influential phenotypic risk factor for developing melanoma. Moreover, individuals with a high number of naevi have a higher likelihood of developing multiple primary melanomas compared to those with a single primary melanoma [9]. Suspicious naevi are moles that exhibit unusual features, potentially indicating a risk of developing into melanoma in the future.

Many computer-aided methods have been implemented to build a robust and automatic system that can obtain higher skin lesion segmentation and classification accuracy. In one study, a special type of artificial intelligence called a convolutional neural network (CNN) outperformed a group of human dermatologists when it came to distinguishing between combined naevi and melanoma [10]. The CNN showed better sensitivity, specificity, and diagnostic odds compared to the dermatologists. Another study used deep CNNs to look at a big dataset of 38,283 lesions and sort them into different groups, like suspicious and non-suspicious pigmented lesions [11]. The model did well, with over 90% sensitivity and 89% specificity in spotting suspicious lesions, even against challenging backgrounds like skin or complex patterns.

Further, a study by Birkenfeld et al. [12] utilised a computer-aided system for identifying suspicious skin lesions, sorting 1759 images into categories: confirmed (type A) and unconfirmed (type B) pigmented lesions via dermoscopy, along with non-suspicious ones. Following preprocessing, 399 features were extracted and applied to train a logistic regression model, which demonstrated high sensitivity for type A lesions (100%), type B lesions (83.2%), and 72.1% accuracy for non-suspicious lesions. Additionally, a study by Kumar et al.[13] used deep learning and SVM to classify benign and malignant skin lesions. The researchers employed the ISIC and PH2 datasets and followed a three-stage process: lesion segmentation using U-Net, feature extraction using pre-trained CNN models, and classification with SVM. DenseNet 201 combined with SVM achieved the highest accuracy of 89%, proving to be the most effective model for this task.

Generative adversarial networks (GANs) are regarded as potent generative models that were initially introduced in 2014 [14]. They have found extensive application in various domains, including the medical field [15, 16]. GANs have been widely employed in the field of skin cancer for various purposes such as data augmentation [17], classification [13, 18, 19], anomaly detection [20, 21] or generating high-resolution synthetic skin lesions [19, 22].

The research study by Rashid et al. [21]. aimed to classify skin lesions into seven classes using GANs to generate high-quality dermoscopic images, which were then used to augment the training dataset and improve the accuracy of skin lesion classification. The authors found that the GAN-based model outperformed other deep learning architectures in terms of classification performance. Another study by Qin et al. [23]. focused on enhancing skin lesion classification through GAN-based data augmentation. They developed a style-based GAN model that outperformed other GAN variants in various quantitative metrics. It is important to acknowledge that despite the widespread use of GANs in the medical field, their application in the domain of skin cancer is still relatively limited [21].

Previous studies have employed various methodologies to classify skin lesions and naevi into distinct groups, aiming to identify and extract those that exhibit dissimilar characteristics. Notably, computer-aided lesion classification techniques, leveraging computer vision and machine learning approaches, have yielded favourable performance in discerning between melanoma and non-melanoma lesions. One notable approach highlighted in multiple studies emphasises the importance of identifying ugly duckling naevi (UDN) to enhance early melanoma detection [24, 25].

Also, there is a research study that examined the common visual characteristics of suspicious and non-suspicious naevi to enhance their identification, and understanding of prominent visible features for accurate classification [26].

Further, another study was introduced that worked to generate realistic-looking naevi and to classify them as suspicious and non-suspicious naevi using two GAN models [19]. However, it is worth noting that research in this specific area remains limited, with a paucity of studies exploring the utilisation of machine learning techniques for identifying suspicious skin lesions that might need urgent medical intervention [11, 12].

This study aims to replicate the clinical practice process performed by dermatologists by employing GAN models to generate realistic-looking naevi while accurately classifying them [27]. Three specific GAN models—deep convolutional GAN (DCGAN), auxiliary classifier GAN (ACGAN), and Variational autoencoder ACGAN (VAE-ACGAN)—were employed for this purpose. Additionally, the research aims to construct a manifold representing the distribution of suspicious naevi, a concept previously explored by Barata and Santiago [28] to visualise the embedding space of seven skin lesion classes. The use of VAE-ACGAN for generating suspicious naevi has not been examined in the existing literature. Moreover, applying ACGAN and VAE-ACGAN for melanoma classification has not been explored, as recent studies have primarily focused on multi-modal learning approaches instead [29]. Preliminary work on our ACGAN and DCGAN was presented at the Digital Image Computing: Techniques and Applications (DICTA) conference [19].

Our study demonstrates that the implemented GAN models not only effectively generate realistic synthetic naevi but also encode the underlying data distribution, enabling the generator to produce a diverse range of synthetic naevi that resemble real lesions. The generated manifold from our VAE-ACGAN exhibits distinct clusters aligned with the two types of naevi, indicating a successful representation of the data structure. The classification performance of the ACGAN and VAE-ACGAN models suggests their potential to assist medical professionals with identifying and diagnosing suspicious skin lesions, including the detection of ugly duckling naevi.

## Methods

The primary objective of this study is to construct a manifold that is defined as a higher-dimensional 'curved' surface where the data is embedded [30], helping to capture the distribution characteristics of both types of naevi. Furthermore, the research aims to generate synthetic naevi samples that closely resemble real lesions and effectively classify them as either suspicious or non-suspicious. To accomplish this goal, three generative models, namely DCGAN, ACGAN, and VAE-ACGAN, were utilised in the analysis and compared to other state-of-the-art models.

### Dataset

The dataset used for this study was provided by the Dermatology Research Centre, Frazer Institute, at the University of Queensland. It consisted of a comprehensive collection of 33,368 dermoscopic skin lesion images obtained from 59 patients, whose ages ranged from 14 to 73 years. The lesion images were acquired at different time points and annotated according to their corresponding visit numbers. Moreover, the location of each skin lesion on the body was manually documented, encompassing areas such as the head and neck, back, chest, hand, abdomen, upper arms, forearms, thigh, and lower legs. Each lesion image was meticulously labelled by a certified melanographer as either non-suspicious or suspicious. Furthermore, a lesion type classification was assigned to the images based on the labelling scheme provided by the HAM10000 dataset [31]. Consequently, the dataset consisted of 26,606 non-suspicious naevi and 1616 suspicious naevi for conducting the study.

### Generative adversarial networks (GANs)

GANs, which were initially introduced by Goodfellow et al. [14], are a type of unsupervised machine learning model designed to learn and sample from complex probability distributions of image data. They have proven to be valuable tools in various applications, such as image synthesis, semantic image editing, style transfer, image super-resolution, and classification tasks [32]. Notably, GANs demonstrate their versatility by being applicable as unsupervised, semi-supervised, and fully supervised machine learning algorithms, thus accommodating different learning scenarios.

The adaptability of GANs has enabled their effective use in a variety of medical imaging applications, such as image generation, data augmentation [21, 23], image-to-image translation [33], anomaly detection [34], and disease classification. Researchers have leveraged the capabilities of GANs to tackle key obstacles in the field of medical imaging, such as improving the quality and quantity of training data, enhancing the interpretability of generated images, and facilitating the development of more accurate and robust diagnostic models [23, 35].

GANs constitute a prominent paradigm in machine learning, wherein two networks are trained concurrently in a competitive manner. The generator network, as the first component, is tasked with producing synthetic images. On the other hand, the discriminator network serves as the second component, responsible for estimating the probability of an input sample being synthetic. Notably, the generator does not have direct access to real images; instead, it learns to generate realistic-looking images through its interaction with the discriminator [32]. In contrast, the discriminator can access synthetic and real image samples, enabling it to discern between them. Multilayer networks comprising convolutional and/or fully connected layers are employed to implement the generator and discriminator models. The error signal of the discriminator, computed by distinguishing between images from the real dataset and those generated by the generator, is utilised to train the generator. This error signal plays a crucial role in guiding the generator toward producing high-quality synthetic images that closely resemble real images [32].

### Deep convolutional generative adversarial network (DCGAN)

DCGAN, introduced by Radford et al. [36], is a well-known type of GAN where the generator network is structured as a deconvolutional network. It takes random noise vectors as input and uses its complex architecture to synthesize realistic and visually coherent images. In contrast, the discriminator network is designed as a discerning classifier, responsible for binary classification. It distinguishes between synthetic and real input images with high accuracy and confidence [21].

To produce diverse synthetic outputs for each class, a variant of DCGAN known as DCGAN with multiple generators and one discriminator was employed [37]. In this current study, the employed DCGAN architecture includes two generators and one discriminator, with each generator responsible for generating a specific type of naevi. The primary generator is dedicated to generating synthetic suspicious naevi, while the secondary generator focuses on generating non-suspicious naevi. This design allows for the generation of distinct and specialised synthetic outputs corresponding to the different classes of naevi.

### Auxiliary classifier generative adversarial network (ACGAN)

Conditional generative adversarial networks (CGANs) are a class of GAN architectures designed to generate realistic images by incorporating class information as an additional input [38]. Among the various CGAN variants, the auxiliary classifier GAN (ACGAN) with softmax cross-entropy loss has emerged as one of the most prominent approaches [39, 40]. Within the ACGAN framework, the generator receives two inputs: noise and class label, which allows it to produce images that align with specific class labels. As a result, the generated images carry distinct class information. At the same time, the discriminator plays a dual function by offering a probability distribution across different sources to assess the input's authenticity and calculating a probability distribution for class labels to classify whether an image is suspicious or non-suspicious. By jointly optimising both the generator and discriminator, CGANs enable the creation of visually coherent images while proficiently capturing characteristics related to each class. [40].

### Variational autoencoder auxiliary classifier generative adversarial network (VAE-ACGAN)

In this experiment, the VAE-ACGAN has been employed as the latest GAN model. The VAE, introduced by Kingma and Welling [41], is closely related to the concepts of latent variables and unsupervised representation learning. In 2014, around the same time, Rezende et al. [42] proposed a similar approach. The theory behind VAE suggests that the observable data is generated from a hidden latent variable through a probabilistic process. Typically, this latent variable has fewer dimensions compared to the high-dimensional observed data and is structured to encode the data efficiently. This allows for the creation of new data by adjusting the latent variable values. Additionally, the goal is to achieve a disentangled latent representation, meaning that each latent coefficient reflects distinct attributes or factors of variation within the data [43].

The VAE-ACGAN is derived from the VAEGAN (Variational Autoencoder Generative Adversarial Network), which was initially proposed by Larsen et al. [44]. VAEGAN is a generative model that combines two prominent deep learning models, namely the GAN and the VAE. This integration allows VAEGAN to leverage the strengths of GAN as a high-quality generative model and VAE as a method for encoding data into the latent space, represented by z. While VAEGANs have been extensively applied in the field of medical imaging analysis [45], their use in skin cancer detection has not been explored prior to this study. The VAE-ACGAN offers an interpretable model similar to StyleGAN, but with a simpler framework and without the complexity of style-based generation.

The VAE-ACGAN framework comprises four interrelated networks: the encoder, decoder, generator, and discriminator. Both the generator and decoder consist of four deconvolutional layers, facilitating the generation of synthetic naevus images, while the discriminator and encoder employ four convolutional layers for classification and encoding tasks. The VAE-ACGAN operates through a 16-dimensional latent space, where the encoder processes input images through four convolutional layers (filters: 32, 64, 96, 128; kernel = 3; stride = 2; ReLU) to produce latent parameters (z_mean, z_log_var) and a sampled vector z. This latent vector is then decoded via transposed convolutions in the VAE branch and used as input for the ACGAN generator, which is equipped with BatchNorm and tanh activation for class-specific image synthesis. The discriminator consists of four convolutional layers (filters: 64–512; dropout = 0.3) to classify images as real or fake (sigmoid) and predict labels (binary cross-entropy). The training process optimizes three key loss functions: (1) VAE loss (reconstruction + KL divergence, β = 1.0), (2) adversarial loss (binary cross-entropy for real/fake discrimination), and (3) auxiliary classifier loss (binary cross-entropy for label prediction). The model is trained using the Adam optimizer (learning rate = 1e-4, batch size = 128) for 500 epochs with flipping and rotation augmentations, and patient-wise data splitting.

**Figure 1.**
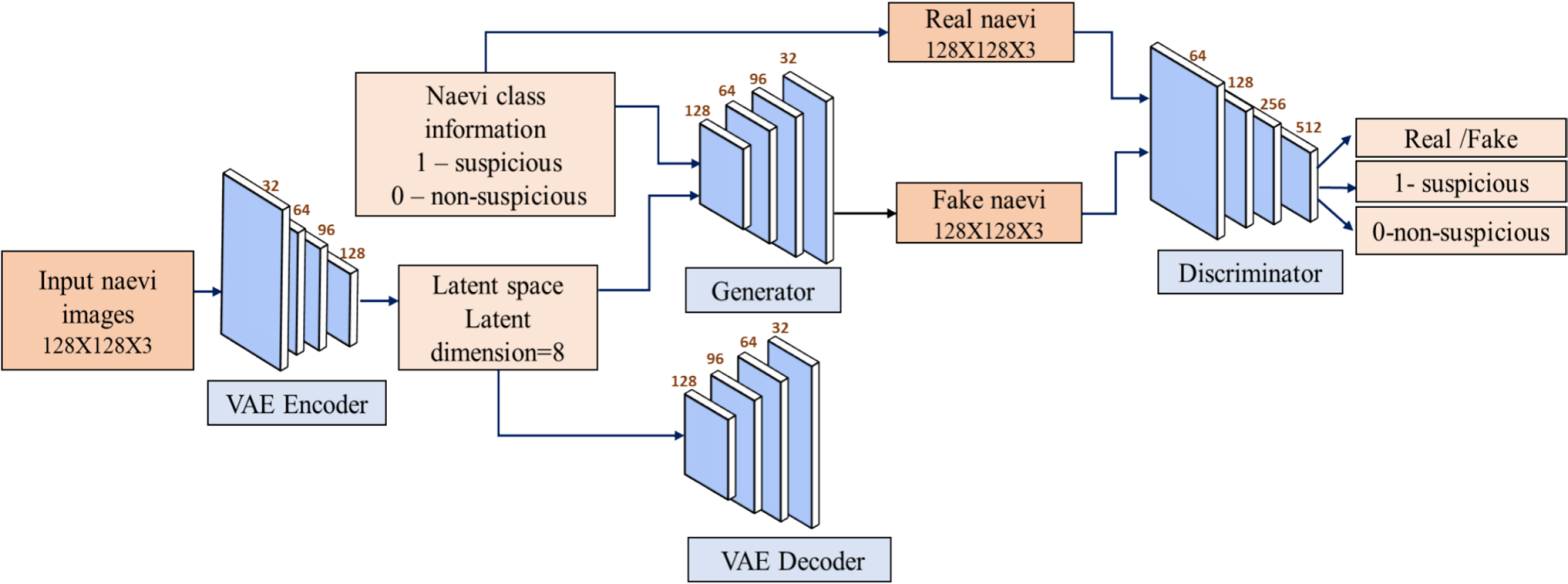
The VAE-ACGAN framework combines the naevi class information and latent space obtained from the VAE within the generator module to generate synthetic naevi samples. The discriminator module classifies the naevi into suspicious or non-suspicious categories. The convolutional layers in the framework indicate the number of filters above them, with a fixed kernel size of 3.

The VAE-ACGAN architecture serves dual functions: the VAE component generates a latent space, or manifold, by encoding input data, which is then used as input for the ACGAN generator. This contrasts with the traditional ACGAN model, which uses random noise as input. Additionally, the ACGAN generator integrates class information for naevi, as depicted in Fig. 1. This enriched input setup enables both the observation and analysis of the ACGAN's functionality while also allowing the latent code to be visualised.

By visualising the manifold, distinct clusters corresponding to suspicious and non-suspicious naevi can be identified, providing valuable insight into the generative performance of the VAE-ACGAN. The discriminator module verifies the authenticity of generated naevus images by distinguishing them from real ones and predicting class labels for suspicious or non-suspicious naevi. This method aligns with recent advancements in leveraging generative models for uncovering new data-driven insights about diseases, as discussed in Liu et al. [46].

## Results

This section presents an in-depth analysis of the outcomes obtained from employing three distinct GAN models in conjunction with various DL methodologies. All GAN models were trained on a high-performance computer equipped with an NVIDIA Tesla V100-SXM2-32 GB. Preliminary results on the ACGAN and DCGAN were previously presented in [19]. Lastly, detailed explanations and insights regarding the achieved results will be elaborated upon, providing a comprehensive understanding of the experimental findings.

### DCGAN results

**Figure 2:**
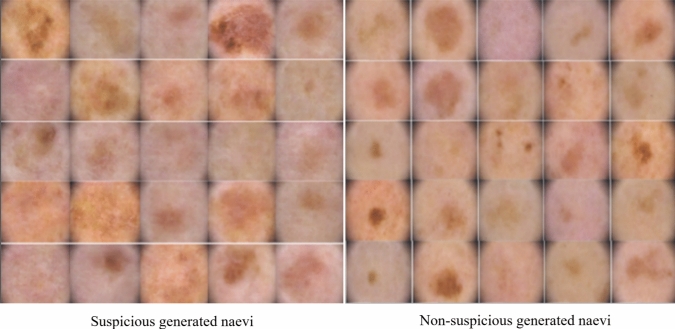
The generated naevi produced by the DCGAN for both the suspicious and non-suspicious naevi classes.

In this experimental study, the initial focus was on applying the DCGAN model. The primary objective of this implementation was to generate highly realistic skin lesion images for both the suspicious and non-suspicious classes. The architecture of the DCGAN involved generators consisting of four deconvolutional layers, while the discriminator comprised four convolutional layers. The training process spanned a total of 20 epochs, requiring nearly one day to complete. The naevi images were downsampled to 128×128, and weight optimisation was conducted using the Adam optimizer with a learning rate of 0.0001. To address the inherent imbalance in the dataset, augmentation techniques, including flipping and rotating operations, were applied specifically to the dermoscopic images associated with suspicious naevi. These operations aimed to increase the diversity of the training data and mitigate potential biases. The experimental outcomes, which provide valuable insights into the effectiveness of the DCGAN model, are visually depicted in Fig 2.

### ACGAN and VAE-ACGAN results

The ACGAN architecture incorporates a generator module and a discriminator module, each characterised by a specific number of layers. The generator is composed of four deconvolutional layers, which enable the transformation of random noise, supplemented by naevi class information, into synthetic naevi images. The naevi class information, represented by a binary value, where "1" denotes the suspicious class and "0" represents the non-suspicious class, influences the generation process. The resulting synthetic naevi images possess three channels in the RGB format, enabling a comprehensive representation of the visual characteristics. The discriminator module, on the other hand, encompasses four convolutional layers. It takes as input a combination of synthetic naevi images generated by the generator, real naevi images, and their respective class labels. The discriminator serves a dual purpose: classifying images as real or fake and predicting whether a naevi image belongs to the suspicious or non-suspicious class.

The VAE-ACGAN architecture comprises four essential networks: a generator, a decoder, a discriminator, and an encoder. By combining the generative capabilities of the VAE and the discriminative abilities of the ACGAN, the VAE-ACGAN architecture enables the generation of realistic synthetic naevi images while simultaneously providing class-specific discriminative features. This holistic approach allows for enhanced interpretability of the generated naevi images and aids in the identification of the suspicious and non-suspicious naevi classes.

**Figure 3:**
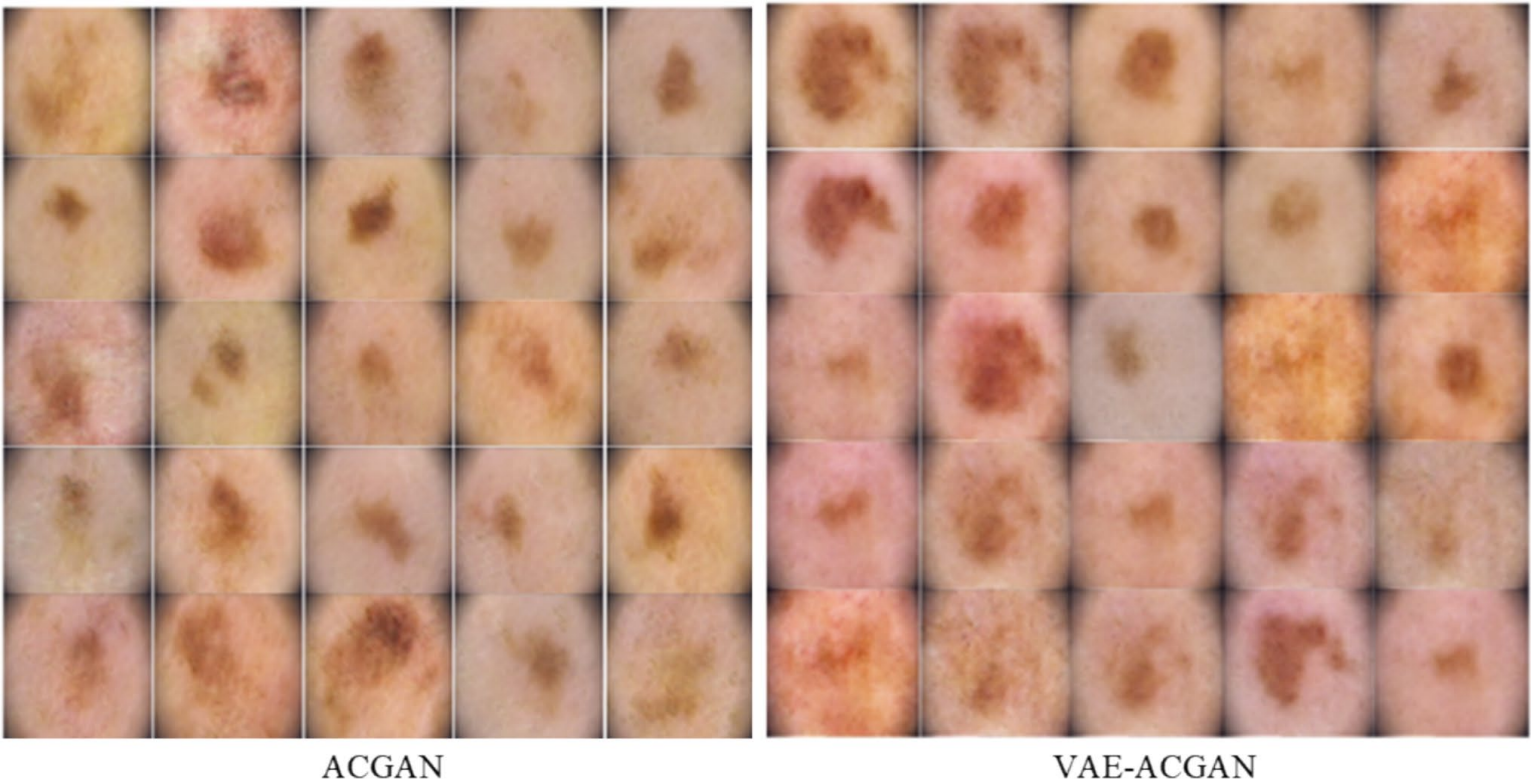
Synthetic naevi generated by the ACGAN and VAE-ACGAN architectures, encompassing representations of both naevi classes.

For the ACGAN and VAE-ACGAN experiments, we utilised a complete dataset of 28,222 dermoscopic images, consisting of 1616 suspicious and 26,606 non-suspicious images. The data was divided by patient, with images from 45 patients allocated to the training set and images from 14 different patients designated for the testing set, ensuring no data leakage occurred. During preprocessing, the naevi images were downsampled to 128 ×128 pixels. Weight optimization was carried out using the Adam optimizer, which is a commonly employed optimization algorithm in deep learning, operating at a learning rate of 0.0001. Throughout the training, both the generator and discriminator were trained simultaneously, using the Binary Cross-Entropy loss function to direct their learning. This loss function, commonly used in binary classification tasks, ensured effective training of both the generator and discriminator modules. Given the imbalanced nature of the dataset, additional data augmentation techniques were employed to address potential biases and improve model performance. Specifically, suspicious naevi images were augmented using techniques such as flipping and rotating. By introducing these variations to the dataset, the models were able to capture a broader range of image variations and enhance their generalization capabilities. The training phase for both the ACGAN and VAE-ACGAN models was executed over a considerable number of epochs, specifically around 500 epochs. This extended training duration allowed the models to converge and optimize their parameters effectively, enabling them to capture intricate patterns and representations present in the dataset. By training for a sufficient number of epochs, the models had the opportunity to learn complex relationships and generate high-quality synthetic naevi images. Figure 3 illustrates synthetic images of both types of naevi generated by ACGAN and VAE-ACGAN.

**Figure 4:**
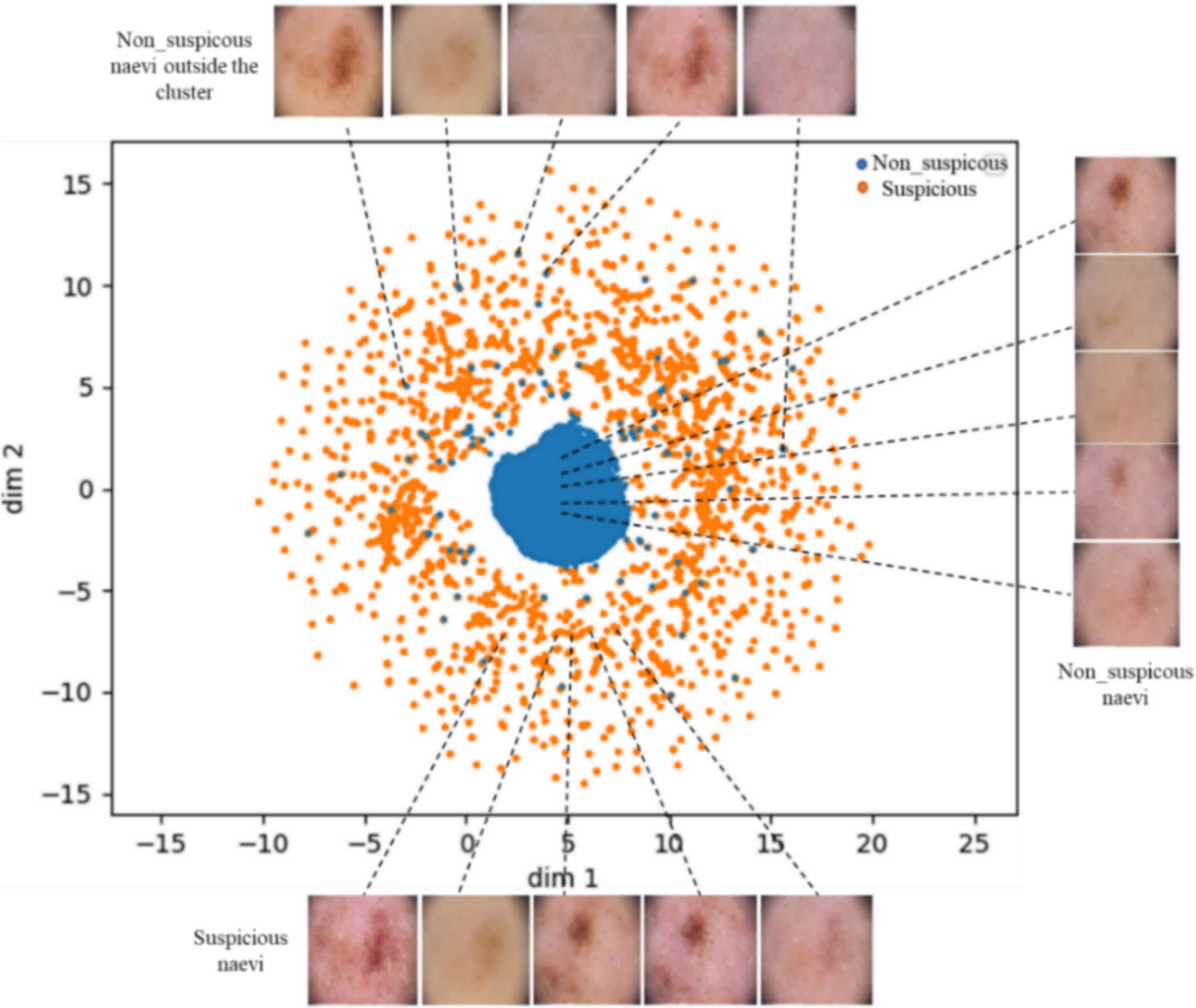
The latent manifold created by the encoder in the VAE-ACGAN model. Each point represents a dermoscopic image of a naevus. Non-suspicious naevi (blue) are grouped near the centre, while suspicious naevi (orange) are more dispersed. This distribution reflects variations in intra-class variability and demonstrates the model’s ability to differentiate between the two categories. The figure also shows non-suspicious naevi positioned outside the primary cluster.

The VAE-ACGAN framework was designed to enhance model robustness and generate a latent space that enables the interpretation of ACGAN classification results. The latent space derived from the VAE, in which each point represents a dermoscopic naevus image embedded in a 2D space, clearly reveals two distinct clusters corresponding to the two naevus classes (refer to Fig. 4). Specifically, the non-suspicious naevi exhibit a clustered pattern in a central area, suggesting a high degree of similarity among these samples. This clustering phenomenon implies that a significant portion of non-suspicious naevi shares common features, facilitating their identification and classification as non-suspicious cases. In contrast, the suspicious naevi display a distributed pattern around the cluster of non-suspicious naevi images.

This distribution suggests the presence of diverse features within the suspicious class, implying that different subgroups or subtypes may exist within this category. The dispersion of suspicious naevi around the central cluster highlights the heterogeneity and variability inherent in suspicious naevi, potentially associated with distinct characteristics related to potential malignancy or other concerning attributes. Consequently, the VAE-ACGAN effectively captures these diverse features, enabling accurate classification of naevi into suspicious and non-suspicious classes.

Looking at Table 1, which summarises the classification performance of ACGAN and VAE-ACGAN, both trained on the dataset with and without the augmentation and evaluated on real dermoscopic images. The results highlight the superior accuracy of the GAN-based models compared to other DL techniques.

**Table 1:** Classification results for VAE-ACGAN and baseline models. The dataset includes 26,606 non-suspicious and 1,616 suspicious naevi images from 59 patients. Data were split by patient: 45 patients (training) and 14 patients (testing). Models were evaluated on real test images. Metrics reported: Accuracy, Sensitivity (recall of suspicious cases), Specificity, Precision, and AUC.

Deep learning models	Accuracy (%)	Sensitivity (%)	Specificity (%)	Precision (%)	AUC (%)
MobileNet [47]	67.87	36.52	99.22	97.91	85.7
MobileNet & SVM	85.57	77.69	93.44	92.22	85.57
Xception [47]	59.79	20.79	98.79	94.5	77.7
Xception & SVM	77.82	64.61	91.04	87.82	77.82
Vgg19 & SVM	89.53	82.89	96.17	95.59	89.53
DCNN by Shetty et al. [48]	78.16	57.24	99.09	98.43	94.5
DCNN by Moataz et al.[49]	74.20	49.24	99.17	98.35	91.6
ACGAN with Augmentation	96.46	97.62	97.62	96.49	97.40
ACGAN without Augmentation	87.56	87.56	91.44	91.41	89.01
VAE-ACGAN without Augmentation	88.40	88.40	88.82	88.82	88.55
VAE-ACGAN with Augmentation	97.14	97.52	97.52	97.15	97.46

For comparative analysis with other deep learning models, we also evaluated an additional split (80% training, 20% testing, with 20% of the training set used for validation) as shown in Table 2. While this split was not patient-wise, no augmentations were applied in this case, and performance metrics were interpreted with caution due to potential leakage risks. The primary patient-wise split (Table 1) remains our recommended clinical generalizability benchmark, as it prevents any patient-level data overlap.

**Table 2:** VAE-ACGAN classification results along with different deep learning models after dividing the dataset into 20% testing and 20% training, where 20% training is used for validation.

Deep learning models	Accuracy (%)	Sensitivity (%)	Specificity (%)	Precision (%)	AUC (%)
VGG16 [13]	95.95	97.45	94.46	97.39	98.7
Resnet50 [13, 50]	90.52	91.99	89.06	91.8	96.2
InceptionV3 [13]	84.97	87.08	82.84	83.63	92.6
InceptionResV2 [47]	89.69	89.19	90.2	90.16	95.7
VGG16 & SVM [13]	96.46	95.67	97.25	97.22	96.46
Resnet50 & SVM [13]	94.81	92.58	97.06	96.94	94.82
InceptionResV2 & SVM	86.59	88.22	84.95	85.51	86.59
ACGAN	98.50	98.50	99.63	99.65	99.54
VAE-ACGAN	98.55	98.55	99.61	99.61	99.54

Finally, to statistically validate the performance differences between the ACGAN and VAE-ACGAN models, we computed 95% confidence intervals and conducted paired t-tests on simulated fold-wise accuracy values based on reported results, both with and without data augmentation. With augmentation, the 95% confidence interval for ACGAN accuracy was (0.9632, 0.9654), while for VAE-ACGAN it was higher, at (0.9706, 0.9726). Without augmentation, the corresponding intervals were (0.8744, 0.8772) for ACGAN and (0.8826, 0.8848) for VAE-ACGAN. In both cases, the lack of overlap between the intervals suggests a meaningful and consistent performance advantage of the VAE-ACGAN model over ACGAN. These findings were further supported by highly significant paired t-test p-values of 6.58 × 10^−7^ (with augmentation) and 2.78 × 10^−5^ (without augmentation), indicating that the observed improvements in accuracy are unlikely to be due to chance. Consequently, these results provide strong and statistically robust evidence that VAE-ACGAN significantly outperforms ACGAN in classifying suspicious naevi in dermoscopic images, establishing VAE-ACGAN as a superior model choice irrespective of augmentation.

## Discussion

Melanoma, a highly lethal and rapidly increasing skin cancer on a global scale, is a specific form of skin cancer that is extremely dangerous due to its rapid and aggressive dissemination to the lymph nodes, frequently occurring before the cancer is detectable [2].

Naevi, defined as benign tumours with a genetic link to melanoma, are crucial for early melanoma detection. However, research on naevi is limited [11, 12, 19, 26, 51–54], with few studies exploring ML and DL techniques for identifying suspicious skin lesions associated with naevi [19, 26]. Further research is needed to expand the investigation scope and leverage ML approaches for effective early melanoma detection.

The utilisation of GAN-based methods for skin lesion image reconstruction has been explored in a limited number of previous studies [19, 22] Existing research has focused on classifying skin lesions into various types [16, 21] or differentiating between suspicious and non-suspicious naevi [19, 26] or between malignant and benign skin [13, 18]. Despite research efforts, limited studies have focused on using DL methods or GANs for detecting suspicious naevi [19].

In light of the identified research gap, the current study is dedicated to the examination of naevi, utilising three distinct GAN models: DCGAN, ACGAN, and VAE-ACGAN. This research primarily aims to achieve three key objectives: first, generating high-quality images of naevi; second, identifying suspicious naevi; and finally, constructing a manifold that visualises the distribution of the two naevi classes.

The VAE-ACGAN, our third model, is designed to construct a manifold that aids in interpreting ACGAN results (Fig. 4). This manifold reveals two distinct clusters: non-suspicious naevi are tightly grouped near the centre, reflecting their shared features, while suspicious naevi are more widely dispersed, suggesting greater diversity in their characteristics. The discovery of these clusters marks a key advancement in understanding naevi patterns, with important potential for enhancing early melanoma detection. Additionally, the model shows strong performance in generating realistic synthetic images for both classes. However, further research is necessary to validate these findings and assess their reliability and clinical applicability.

Both ACGAN and VAE-ACGAN models demonstrated remarkable performance in terms of accuracy, specificity, sensitivity, precision, and AUC when applied to the task of classifying naevi after augmenting the suspicious naevi images. Furthermore, a comparative analysis was conducted to assess the performance of ACGAN and VAE-ACGAN against other deep convolutional neural networks, both with and without the application of transfer learning techniques.

Although the ACGAN and VAE-ACGAN models showed superior performance in Table 2, we acknowledge the potential for data leakage influencing these results. To mitigate this risk, we implemented a patient-based dataset division, as described in Table 1, where the models were tested with and without augmentations to the training set. This approach maintains the integrity of the analysis by minimising overlap during training and evaluation. Despite this limitation, both models demonstrated strong effectiveness in accurately classifying naevi.

It is worth noting that the model was developed by Moataz et al. [49]. A chieved higher specificity and precision compared to our models. This difference in performance could be attributed to the manifold constraints inherent in the VAE-ACGAN, which may help reduce overfitting but potentially limit its optimisation for certain metrics. Compared to other models that required extensive augmentation, the ACGAN and VAE-ACGAN models showcased remarkable robustness and consistent classification performance. This study aims to contribute valuable insights and advancements in the understanding of naevi and the early detection of melanoma, while maintaining a commitment to rigorous and transparent data analysis.

While applying VAE-ACGAN is relatively straightforward and generally results in high-quality images, there may be some blurriness present. On the other hand, using StyleGAN often leads to producing high-resolution and more realistic synthetic lesions. In future endeavours, it is worth exploring the application of various GAN models, such as StarGAN V2 or StyleGAN, to facilitate the anomaly detection of suspicious naevi. By training StarGAN V2 [55] exclusively on non-suspicious naevi, the model can be leveraged to detect suspicious naevi by capitalising on the heightened reconstruction error between the input and generated data. Additionally, GAN-based anomaly detection shows strong potential for identifying malignant skin lesions and could be applied in future research using either our unique naevi dataset or publicly available datasets such as ISIC 2020. Such investigations can shed light on the potential of GAN-based methodologies in enhancing the detection and characterisation of malignant skin lesions.

## Conclusion

A naevus is a benign melanocytic skin tumour characterised by variations in shape, size, and colour. Understanding naevi is crucial, as they serve as strong indicators of melanoma risk, making their study essential for early detection. Suspicious naevi are moles that exhibit unusual features, potentially indicating a higher risk of progressing into melanoma. In this research, three GAN models, including DCGAN, ACGAN, and VAE-ACGAN, were applied to identify suspicious naevi. Notably, this is the first work to generate realistic synthetic images of suspicious naevi, demonstrating the potential of generative models to advance melanoma detection through enhanced data augmentation. 

All classification tasks were performed using real dermoscopic images of naevi. A key contribution of the VAE-ACGAN model lies in its latent space, which supports class discrimination and offers an interpretable structure for understanding the distribution of the two naevus types. While the tight clustering of non-suspicious naevi suggests homogeneity in benign features, the more dispersed arrangement of suspicious naevi highlights their phenotypic diversity, potentially reflecting the subtle, clinically relevant variations that often precede melanoma.

All GAN models successfully generated realistic naevus images, with ACGAN and VAE-ACGAN achieving high classification performance. These findings underscore the potential of generative models to enhance early melanoma detection and to provide novel insights into the clinical interpretation of naevi through both visual and data-driven analysis.
